# DNA 5mC methylation inhibits the formation of G-quadruplex structures in the genome

**DOI:** 10.1186/s13059-025-03678-4

**Published:** 2025-07-11

**Authors:** Kangkang Niu, Lijun Xiang, Xiaojuan Zhang, Xiaoyu Li, Tingting Yao, Jin Li, Chu Zhang, Junpeng Liu, Yuling Peng, Guanfeng Xu, Hui Xiang, Hao Wang, Qisheng Song, Qili Feng

**Affiliations:** 1 Guangdong Provincial Key Laboratory of Insect Developmental Biology and Applied Technology, Guangzhou Key Laboratory of Insect Development Regulation and Application Research, Institute of Insect Science and Technology, School of Life Sciences, South China Normal University, Guangzhou, 510631 China; 2 Guangdong Laboratory for Lingnan Modern Agriculture, Guangzhou, China; 3 South China Normal University-Panyu Central Hospital Joint Laboratory of Basic and Translational Medical Research, Guangzhou Panyu Central Hospital, Guangzhou, China; 4 Division of Plant Sciences and Technology, University of Missouri, Columbia, MO 65211 USA

**Keywords:** DNA methylation, G-quadruplex formation, Epigenetic regulation, Gene expression

## Abstract

**Background:**

G-quadruplex structures (G4s) have been identified in the genomes of many organisms and have been proven to play significant epigenetic regulatory roles in gene transcription. Intriguingly, only a small portion of the predicted G4-forming sequences can fold into G4s under cellular conditions. Here, we employ publicly available data, methylation inhibitors, DNA methyltransferase 1 (DNMT1) knockout, and multiple ‘Omics’ technologies to study the interplay between DNA methylation and chromatin accessibility on G4 formation and the impact on gene expression.

**Results:**

We find an antagonistic correlation between genomic 5mC DNA methylation level and G4 abundance. The abundance of genomic G4s significantly increases when the genome-wide methylation level is reduced by methylation inhibitor treatment or DNMT1 knockout. The increase in G4 signals in DNMT1 knockout cells is reversed by DNMT1 overexpression. Combined ATAC-seq, whole genome bisulfite sequencing, and G4 CUT&Tag analyses demonstrate that 5mC DNA methylation inhibits G4 formation in both open and closed chromatin states. The inhibitory effect of 5mC modification on the formation of G4s is verified by circular dichroism and electrophoretic mobility shift assay in vitro. G4 CUT&Tag and RNA-seq analyses reveal that reduced DNA methylation enhances G4 formation and promotes the transcription of nearby genes.

**Conclusions:**

This study demonstrates that 5mC DNA methylation directly inhibits G4 formation in the genome and modulates subsequent gene transcription, confirming the interaction between G4s and DNA methylation as an important mechanism for epigenetic regulation of gene transcription.

**Supplementary Information:**

The online version contains supplementary material available at 10.1186/s13059-025-03678-4.

## Background

G-quadruplex structures (G4s) are tetraplex structures typically formed in guanine-rich sequences [1–3]. Four guanines are mutually connected by Hoogsteen hydrogen bonds to form a G-tetrad, and two or more G-tetrads stack together into a G4 [4–6]. G4s are prevalent in various organisms [7] and have been found to play critical roles in multiple cellular activities, including telomere protection, DNA replication, gene transcription, and protein translation [8–10]. In particular, G4s are enriched in gene transcription regulatory regions and regulate gene transcription [11–13], especially that of oncogenes such as c-MYC, KRAS, and BCL2 [14–17]. Dynamic gene transcription regulated by G4s implied that G4s are formed in a spatiotemporal manner, which could account for the discrepancy that the number of actually formed (detected) G4s is much less than the number of predicted structures [18, 19] and that G4s were abundant in embryonic stem cells but are lost during lineage specification [20]. Besides, dysregulated G4 formation were found in liver and stomach cancer tissues [21]. All these indicate us to study the G4 formation mechanism, which is critical for elucidating the function of G4 on transcription of specific genes and further related diseases.

The dynamic formation of G4s could be influenced by multiple factors, such as monovalent cations [22], molecular crowding [23], G4-binding proteins [24–27], chromatin accessibility [28], and epigenetic modifications [29]. DNA methylation at the 5-position of cytosine (5mC) is a central regulator of gene expression and chromatin structure [30, 31]. The enrichment of PQSs in CG-rich regions, which are also methylation sites, suggests functional relationships between G4 and DNA methylation. In our previous study, an antagonistic relationship between PQSs density and DNA methylation was found in *Sus scrofa domestic* and *Bombyx mori* genomes [32]. Similar observations were also found in human cells, G4 peaks were largely overlapped with CpG island (CGI) and were associated with hypomethylation [33, 34]. This negative correlation could be interpreted by the fact that formed G4 structure could protect CGI from methylation through binding with DNMT1 and inhibiting its enzymatic activity [33]. Nevertheless, there is still a lack of explanation for this antagonistic from the perspective of the role of DNA methylation on G4 formation. Some in vitro evidence with individual genes revealed dual effects of DNA methylation on G4s stability. The methylation of CpG regions increases the stability of G4s in the BCL2 and C9orf72 promoters [35, 36], while destabilize all three G4s in MEST gene promoter [29]. Circular dichroism (CD) spectrum analysis of methylated G4 sequences of VEGF, HRAS2, and c-kit revealed a lower G4-specific absorption peak at 260 nm than that of unmethylated G4 sequences, and methylated HRAS1 G4 showed non-G4 characteristics [37]. Although these studies indicate that DNA methylation may has important effects on G4 formation, the genome-wide intrinsic relationship between DNA methylation and G4 formation still needs to be further clarified.

Here, we investigated the genome-wide relationship between DNA methylation and G4 formation by utilizing the publicly available genomic data, DNMT1 knockout cell lines, and the treatment of DNMT1 inhibitors. Through multiple omics analyses, an antagonistic relationship was revealed for these two epigenetic regulators of gene transcription. DNA methylation at CpGs within promoter PQSs inhibited the formation of G4s in both open and closed chromatin contexts and then influenced gene transcription. This work provides new insight into the interaction and coordination of these two epigenetic factors in gene transcription that DNA methylation could regulate gene transcription by affecting the formation of promoter G4s.

## Results

### G4 formation is negatively correlated with DNA 5mC methylation

To investigate the effect of DNA methylation on the formation of G4s, whole-genome bisulfite sequencing (WGBS) and G4 CUT&Tag data from human HeLa and K562 cancer cell lines were obtained from NCBI, and the correlation between these two signals was analyzed.

**Fig. 1:**
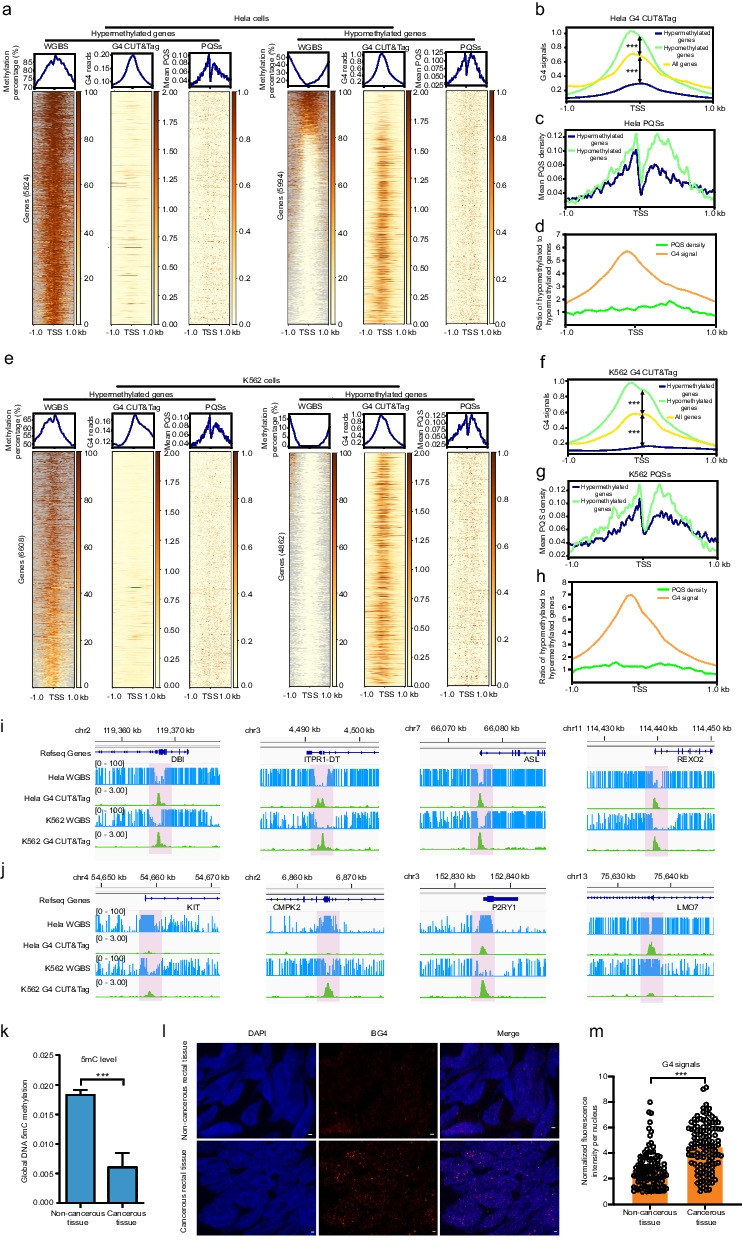
Comparison of DNA methylation and G4 formation around the TSSs of human genes. **a**, **e** Heatmap analysis of methylation, G4s and PQSs in HeLa and K562 cells. **b**, **f** The distribution of G4s in the 1 kb upstream and downstream regions around the TSSs of genes. **c**, **g** The distribution of PQSs in hypermethylated and hypomethylated genes. **d**, **h** The ratios of PQS density and G4 signal density of hypomethylated to hypermethylated genes. **i** Integrative Genomics Viewer (IGV) screen shots showing the coincidence of G4 peaks (green) with hypomethylated sites in HeLa and K562 cells. The whole genome bisulfite sequence (WGBS) tracks are shown in blue. **j** IGV screen shots showing that in HeLa cells, the KIT, CMPK2, and P2RY1 sites had high methylation levels and low G4 enrichment, while the LMO7 sites had low methylation levels and high G4 enrichment. In K562 cells, KIT, CMPK2, and P2RY1 methylation decreased, and G4 enrichment increased, whereas LMO7 methylation increased, and G4 enrichment decreased. **k** Methylation level in cancerous rectal tissue and noncancerous rectal tissue. **l**, **m** The detection and quantitative analysis of G4s in cancerous rectal tissue and noncancer rectal tissue. The data shown are mean ± SEM. Statistical significance was determined by Student’s *t* test, ***p < 0.001. The scale bars equal 1 μm

Transcripts were ordered by the degree of methylation in the regions between 1 kb upstream and downstream of transcription start sites (TSSs). After selecting a single transcript per gene, 5824 hypermethylated and 5994 hypomethylated loci were obtained from the top and bottom 10,000 genes for subsequent analyses. Next, the G4 signals for the 1 kb upstream and downstream regions around the TSSs for the same transcripts were extracted and compared to the methylation signal. G4 signals were significantly lower for hypermethylated genes and notably greater for hypomethylated genes (Fig. 1a, b, e, f). To exclude the possible influence of sequence content variance on the above analyses, PQS abundance in both hypermethylated and hypomethylated genes was analyzed. The results revealed a slight increase in PQS abundance for hypomethylated genes, especially upstream of the TSSs where G4 CUT&Tag peaks are enriched (Fig. 1c, g). The ratio of the PQS density for hypomethylated genes to that for hypermethylated genes ranged from 0.719 to 1.875 in Hela cells and 0.643 to 1.593 in K562 cells, while the G4 signal density for hypomethylated genes was much greater than that for hypermethylated genes (1.679–5.719 fold in Hela cells and 1.329–7.009 fold in K562 cells) (Fig. 1d, h). Although PQS abundance was similar between these two sets of hypomethylated and hypermethylated genes, fewer G4 signals were detected in hypermethylated genes due to the inability of PQSs to form G4s, not due to the low content of PQSs. These results strongly implied a negative relationship between the DNA methylation level and G4 formation in the human genome. Additionally, visual alignment of DNA methylation levels and G4 signals in both HeLa and K562 cells showed that G4 enrichment was negatively correlated with methylation levels in eight random PQS-containing genes (Fig. 1i, j). For the genes *DBI*, *ITPR1-DT*, *ASL*, and *REXO2*, which showed consistent G4 abundances and methylation levels in both cell lines, higher G4 enrichment in the PQS region was always associated with lower methylation levels (Fig. 1i). Similarly, for the genes *KIT*, *CMPK2*, *P2RY1*, and *LMO7*, although the methylation levels were different between HeLa and K562 cells, the antagonistic relationship between G4 enrichment and methylation level was also obvious. For *KIT*, *CMPK2*, and *P2RY1*, a high methylation level in HeLa cells resulted in low G4 signals, whereas a low methylation level in K562 cells resulted in greater G4 signals (Fig. 1j). Likewise, for *LMO7*, a low level of methylation in HeLa cells resulted in a high level of G4 enrichment, and a high level of methylation in K562 cells resulted in a low abundance of G4s (Fig. 1j).

A reduced level of genome-wide methylation is a typical feature of oncogenesis [38–42] and allows to test the negative correlation between DNA 5mC methylation and G4 quantitatively at a global level. Cancerous and noncancerous rectal tissues were isolated from patients and confirmed with histochemistry for cancer markers. The proliferation of cancerous tissues was also verified by Ki-67 staining, and noncancerous tissues were used as controls (Additional file 1: Fig. S1). ELISAs with an anti-5mC antibody showed that the overall genomic methylation level in the cancerous rectal tissue was significantly lower than that in the noncancerous rectal tissue (Fig. 1k); moreover, immune-fluorescence (IF) experiments with an G4-specific antibody showed that the abundance of genomic G4s in cancerous tissues was significantly greater than that in the noncancerous tissues (Fig. 1l, m).

Taken together, these findings clearly revealed a negative correlation between DNA 5mC methylation and G4 formation at the genomic level. The finding of a negative correlation between G4 and 5mC methylation is consistent with previous studies [20, 32, 33, 43, 44].

### G4 abundance significantly increases when methylation is suppressed

**Fig. 2:**
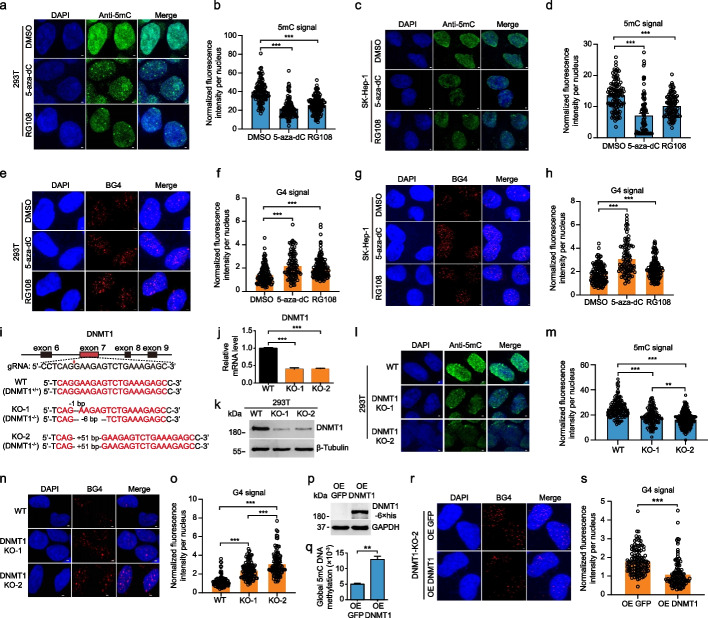
Effect of reduced methylation levels on G4 formation.** a**, **e** Detection of methylation levels by IF in cells treated with 5-aza-dC or RG108. **b**, **f** Variation in G4 formation detected by IF using BG4 in cells treated with 5-aza-dC or RG108. **c**, **d**, **g**, **h** Quantitative statistical analysis of the methylation level and G4 formation in cells treated with 5-aza-dC or RG108. **i** Design of the knockout target sites and sequencing confirmation of the identified mutant strains. **j**, **k** mRNA and protein levels of the *DNMT1* gene in wild type and mutant cells. **l**–**o** Detection and quantitative analysis of methylation levels (**l** and **m**) and G4 signals (**n** and **o**) in wild type and mutant cells. **p** Assessment of DNMT1 expression by western blot. **q** Changes in the methylation level after the exogenous re-expression of DNMT1 in DNMT1-KO-2 cells. **r** Assessment of the G4 signal after the exogenous re-expression of DNMT1 in DNMT1-KO-2 cells. **s** Quantitative analysis of the IF in **r**. The data are presented as mean ± SEM. Statistical significance was determined by Student’s *t* test, **p < 0.01, ***p < 0.001. The scale bars equal 1 μm

DNMT inhibitors, such as 5-Aza-dC and RG108, have been used previously to study the role of DNA methylation. 5-Aza-dC is a cytosine analog that blocks DNMT function by covalently binding to the catalytic cysteine residue of DNMT [45]. RG108 is a nonnucleoside inhibitor that acts by reversibly blocking the active site of DNMT without incorporation into the DNA strand [46]. We treated 293 T and SK-Hep-1 cells with 5-Aza-dC and RG108 to reduce the global methylation level and then studied the effect on G4 formation. Methylation levels and G4 abundance were measured using IF assays. In 293 T cells as well as SK-Hep-1 cells, the methylation level significantly decreased when the cells were treated with either 5-aza-dC or RG108 (Fig. 2a–d), while at the same time the G4 signals notably increased (Fig. 2e–h). As before, the reduction in the genomic methylation level (induced by methylation inhibitors) enhanced the formation of G4s.

Extensive studies have shown that the whole-genome methylation level is significantly reduced when DNMT1 is knocked out [47, 48]. In this study, DNMT1 was knocked out in 293 T cells by using CRISPR/Cas9 gene editing, and the genomic methylation and G4 signal levels were assessed. Specific sgRNAs were designed to target exon 7 of the DNMT1 gene (Fig. 2i) and were transfected into 293 T cells along with a Cas9 plasmid to achieve DNMT1 knockout. Two mutant cell lines, DNMT1-KO-1 and DNMT1-KO-2, were obtained after monoclonal cell screening and confirmed by genomic DNA sequencing. In the DNMT1-KO-1 cell line, the DNMT1 alleles were mutated in an asymmetric manner: one allele had a 1-bp deletion, leading to an open reading frame shift, and the other allele had a 6-bp deletion, resulting in the absence of two amino acid residues compared to the wild type (Fig. 2i). In the DNMT1-KO-2 cell line, both DNMT1 alleles received a 51-bp insertion at the same position, resulting in the addition of 17 amino acid residues (Fig. 2i). Compared to the wild-type cell line, the expression of DNMT1 in both mutant cell lines was significantly decreased at both the mRNA and protein levels (Fig. 2j, k). IF assays showed that the overall methylation level was notably reduced in both mutant cell lines, especially in the DNMT1-KO-2 cell line (Fig. 2l, m), while the G4 signals were significantly increased. The effect was stronger in the DNMT1-KO-2 cells than in DNMT1-KO-1 cells (Fig. 2l–o). These results suggested that DNMT1 mutation resulted in a decrease in overall methylation in the genome, which then led to a significant increase in the abundance of genomic G4s.

Finally, we expressed the recombinant DNMT1-6 × his protein in DNMT1-KO-2 cells and assessed the reconstituted genomic methylation level and its effect on G4 formation. Western blots showed that DNMT1-6 × his was successfully expressed in the DNMT1-KO-2 cells (Fig. 2p) and the overall methylation level was significantly higher compared to the control (DNMT1-KO-2 cells transfected with the EGFP vector) (Fig. 2q), Again, the increased methylation levels were accompanied by a significant reduction in genomic G4 signals (Fig. 2r, s). The results of these experiments further confirmed the antagonistic correlation between methylation and G4 formation in the genome. A low level of DNA methylation is beneficial for the formation of genomic G4s.

### Genomic hypomethylation enhances G4 formation partly independent of altered chromatin accessibility

**Fig. 3:**
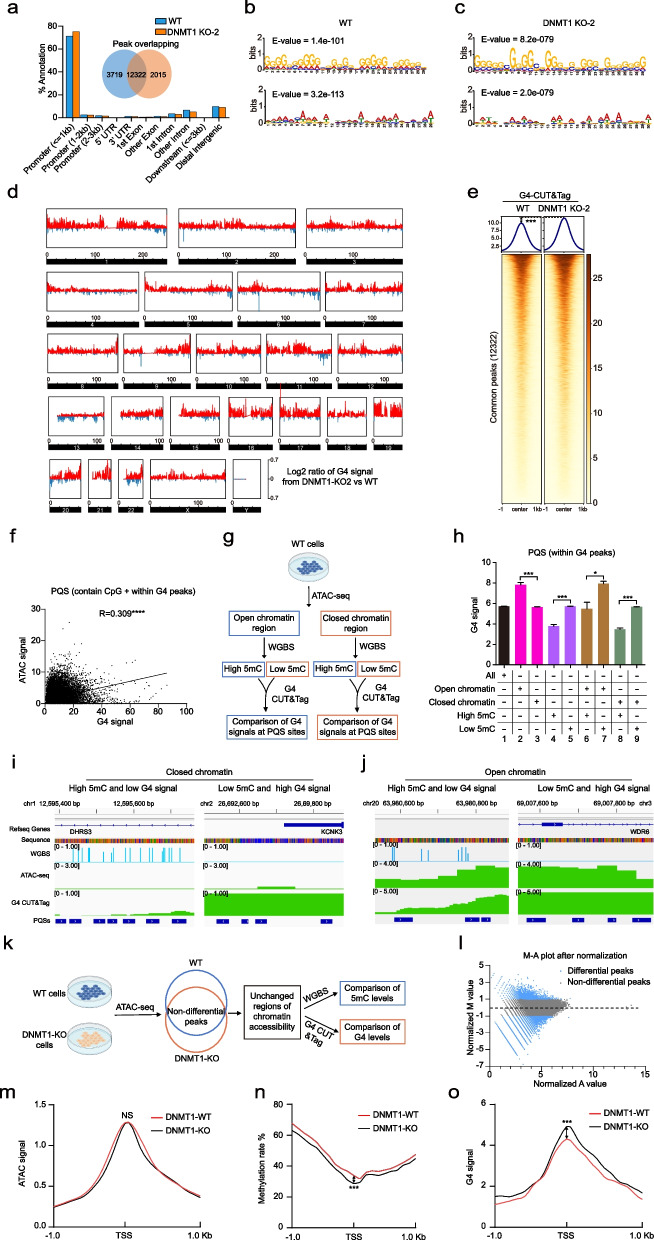
Effect of methylation and chromatin accessibility on G4 formation.** a** Genome-wide annotation of 293 T WT and DNMT1-KO-2 G4 CUT&Tag peaks, presented as percentages. Venn diagram showing the number of WT- or DNMT1-KO-2-specific peaks and overlapping peaks. **b**, **c** MEME-chip analysis of the BG4 peaks of WT or DNMT1-KO-2 cells, showing the top two motifs. **d** Changes in G4 signal of each chromosome in DNMT1-KO cells compared to WT cells. The values represent the log2 ratio of DNMT1-KO-2 vs. WT cells. For the DNMT1-KO-2 and WT BG4 signals, IgG signals were used as the background, and the signals were normalized to the bins per million mapped reads (BPM). **e** Heatmap of G4 CUT&Tag enrichment for WT or DNMT1-KO-2 cells centered on common peaks (12,322) ± 1 kb in length. **f** Correlation of G4 with chromatin accessibility. The signals of the 56,415 PQSs (with a G4 peak and containing CpG) were counted and calculated by Pearson’s correlation. **g** Schematic for analyzing the effect of chromatin accessibility and methylation on G4s in 293 T cells. **h** G4 signals at different chromatin accessibility or methylation rate sites. The signals of the 56,415 PQSs were counted. The PQSs overlapping with ATAC peaks are considered open chromatin PQSs, and those containing CpG sites with a methylation rate greater than 50% are considered high-methylation PQSs. **i, j** IGV screen shots showing the WGBS signal (blue), ATAC signal (green), and G4 signal (green) in closed chromatin and open chromatin regions. **k** Schematic for analyzing the effect of chromatin accessibility and methylation on G4s in WT and DNMT1-KO cells. **l** Identification of regions with similar chromatin accessibility between WT and DNMT1-KO cells. **m** Comparison of ATAC signals in similar regions between WT and DNMT1-KO cells. **n**, **o** Analysis of the methylation rate and G4 signals in regions with similar chromatin accessibility. The data are presented as mean ± SEM. Statistical significance was determined by Student’s t test, *p < 0.05, ***p < 0.001, ****p < 0.0001. NS represents non-significance

Given that chromatin accessibility is an important factor affecting G4 formation [28], we next investigated whether the decreased G4 formation in hypermethylated regions was due to DNA methylation inhibition alone or additionally associated with decreased chromatin accessibility. We performed G4 CUT&Tag (for G4 signals), ATAC-seq (for chromatin accessibility), and WGBS (for methylation) analyses in the 293 T cell line (WT) and the derived cell line DNMT1-KO-2. In total, 16,041 and 14,337 G4 peaks were identified in the WT and DNMT1-KO-2 cells, respectively. Venn diagram distribution analysis revealed 12,322 common peaks in both WT and DNMT1-KO-2 cells (Fig. 3a). G4 peaks were mainly localized in gene promoter regions and more abundant in DNMT1-KO-2 cells (75.3%) than in WT cells (71.3%) (Fig. 3a). Moreover, motif discovery revealed that the top two statistically significant motifs possessed classical G4 characteristics and were almost identical between WT and DNMT1-KO-2 cells (Fig. 3b, c). The whole-genome BG4 signals were significantly greater in the DNMT1-KO-2 cells than in the WT cells (Fig. 3d). Furthermore, the intensity of the 12,322 common G4 peaks in DNMT1-KO-2 cells was also greater than that in WT cells (Fig. 3e), indicating that more G4s formed in the hypomethylated cells in which DNMT1 was knocked out. Within the 293 T cells, we identified 56,415 PQSs that contained a G4 peak and at least one CpG dinucleotide. Correlation analysis of the ATAC signal and the G4 signal closed to the PQS-containing CpG sites was conducted. The results showed that G4 signals were weakly positively correlated with ATAC signals (Pearson correlation coefficient R = 0.309) (Fig. 3f), which was consistent with the findings of Li et al. [13]. The low correlation coefficient between G4 and ATAC signals suggested that in addition to chromatin accessibility, other more critical factors may influence G4 formation in the genome. As a previous report pointed out, additional mechanisms may influence G4 formation in NHEK cells excepting the nucleosome-depleted environment [19]. We further analyzed the effects of both chromatin accessibility and methylation level on G4 formation in 293 T cells (Fig. 3g). G4 signals in open chromatin regions were greater than the G4 signals in the closed chromatin regions when considering only chromatin accessibility (Fig. 3h, columns 2 and 3). When considering only DNA methylation level, G4 signals in hypomethylated PQSs were significantly greater than those in hypermethylated PQSs (Fig. 3h, columns 4 and 5). Considering both chromatin accessibility and methylation levels, more G4s formed in hypomethylated regions in both open and closed chromatin regions (Fig. 3h, columns 6–9; Fig. 3i, j).

Next, we compared the 293 T wild type cells with the DNMT1-KO-2 cells for chromatin accessibility, methylation, and G4 signals (Fig. 3k). First, we identified non-differential ATAC peaks between both cell lines using the MAnorm software [49], and then selected genes that contained ATAC peaks in their promoter regions (approximately TSS ± 1 kb) for further analysis (Fig. 3l). Statistical analysis revealed no differences in the ATAC signals between WT and DNMT1-KO-2 cells (Fig. 3m). However, DNA methylation levels in promoter regions of these genes were lower in DNMT1-KO-2 cells than in WT cells, and correspondingly, G4 signals were notably greater in DNMT1-KO-2 cells than in WT cells (Fig. 3n, o). Together, these results confirm that hypomethylation enhances G4 formation without the need for open chromatin, but G4 formation in hypomethylated promoter regions is further promoted by increased chromatin accessibility.

### Genomic hypomethylation enhances gene transcription by increasing G4 formation

**Fig. 4:**
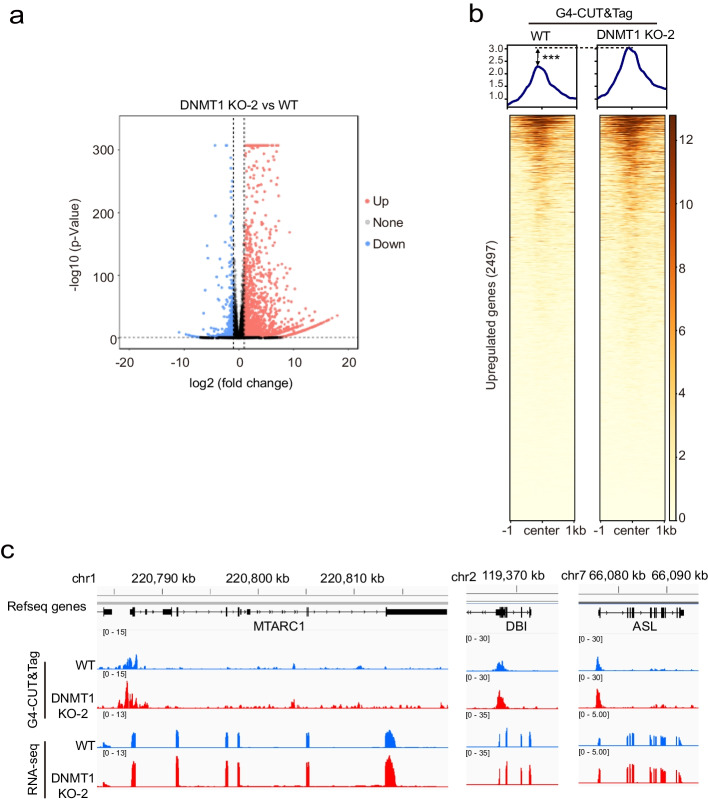
Hypomethylation enhances gene transcription by increasing G4 formation.** a** Volcano plot for the comparison of RNA-seq data between WT and DNMT1-KO-2 cells. Genes whose expression did not change are shown in black, while genes whose expression differed (fold change > 2 and *p*-value < 0.05) are denoted in blue or red. **b** Heatmap of G4 CUT&Tag enrichment of promoter regions (TSS ± 1 kb) of upregulated genes (2497) in DNMT1-KO-2 vs. WT cells. **c** Genome browser tracks showing the distribution of G4s and RNA-seq data for the WT (blue tracks) and DNMT1-KO-2 (red tracks) cells. The black track and arrows at the top show the gene structures (5′UTR, exons, introns, 3′UTR) and the direction of transcription. The data are presented as mean ± SEM. Statistical significance was determined by Student’s *t* test, ***p < 0.001

It has been reported that gene transcription is associated with G4 formation in promoter regions [19] and hypomethylation in mammals [50]. Therefore, we analyzed whether hypomethylation in the human genome affects gene transcription through G4 formation using RNA-seq. Differential expression analysis between the 293 T cells and DNMT1-KO-2 cells revealed 3647 differentially expressed genes (DEGs) among which 2645 genes were upregulated and 1002 genes were downregulated when DNMT1 was knocked out (Fig. 4a). We analyzed the G4 peaks located in the promoter region of the upregulated DEGs (2497 out of 2645 genes had well-defined TSSs) and found that the G4 signals near the TSSs (± 1 kb) of the upregulated DEGs increased in the DNMT1-KO-2 cells (Fig. 4b). For example, the MTARC1, DBI, and ASL genes had a greater abundance of G4s in their promoter regions and more RNA-seq reads in DNMT1-KO-2 cells than in WT cells (Fig. 4c). Taken together, these results demonstrated that a reduction in global genome methylation enhanced the formation of G4s, which then increased the global transcription level.

### 5mC DNA methylation directly prevents folding of PQSs into G4s

**Fig. 5:**
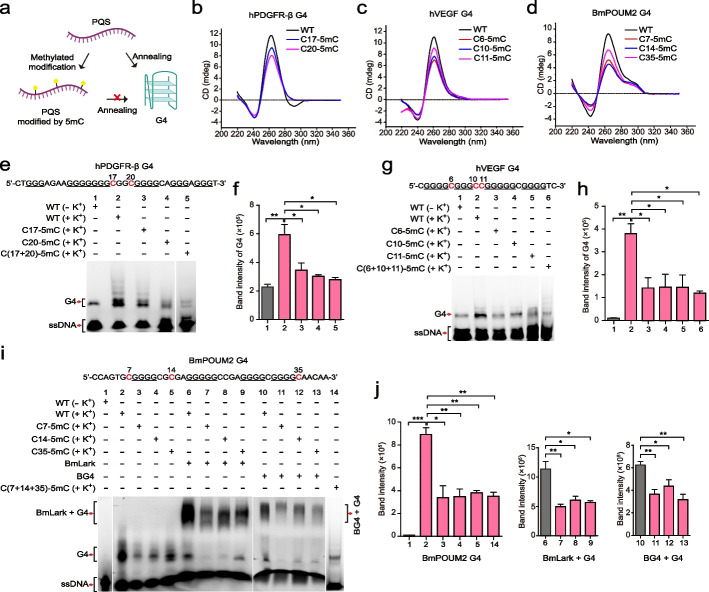
The effect of 5mC modification on G4 formation.** a** Descriptive diagram of the probe treatment method used for the CD analysis and EMSA. **b**–**d** CD analysis of the effect of 5mC modification on G4 formation. **e**, **g, i** EMSA of the effect of 5mC modification on G4 formation and G4 binding to proteins. **f**, **h**, **j** Quantitative statistics of the effect of 5mC modification on G4 formation and G4 binding to proteins. The data are presented as mean ± SEM. Statistical significance was determined by Student’s *t* test, *p < 0.05, **p < 0.01, ***p < 0.001

We analyzed three well-studied PQSs located in the promoters of the *hPDGFR-β* [51], *hVEGF* [52] and *BmPOUM2* genes [24, 53] to further confirm the inhibitory effect of 5mC modification on G4 formation. These PQSs have been experimentally proven to be capable of forming G4s either in vitro or ex vivo. The methylation sites in the G4 region of the *BmPOUM2* promoter were determined by bisulfite sequencing PCR (BSP) analysis (Additional file 1: Fig. S2a). The methylation sites in the G4s of the *hPDGFR-β* and *hVEGF* promoters were identified from whole-genome bisulfite sequencing (WGBS) data (Additional file 1: Fig. S2b, c). Two 5mC modification sites are located in the same loop of the *hPDGFR-β* promoter G4, while three 5mC sites are located in two loops of the *hVEGF* promoter G4, and three 5mC sites are distributed in the 5′ end, first loop, and 3′ end of the *BmPOUM2* promoter G4 (Additional file 1: Fig. S2). Oligonucleotides of the selected G4s of these genes with or without 5mC modification were synthesized and used for CD analysis (Fig. 5a). The results showed that the G4 feature peak at 265 nm was lower for all the 5mC-modified oligonucleotides than for the unmodified oligonucleotides (Fig. 5b–d), indicating that 5mC methylation suppressed the formation of G4s in these oligonucleotides. This inhibitory effect of 5mC methylation on G4 formation was further confirmed by electrophoretic mobility shift assay (EMSA). More G4s formed in the PQS oligonucleotides of all three genes in the presence of K^+^ than in the absence of K^+^ (Fig. 5e–i, lanes 1 and 2). The G4 band intensity substantially decreased or even disappeared when these PQS oligonucleotides were methylated (Fig. 5e, f, lanes 3–5; 5 g, h, lanes 3–6 and 5i, j, lanes 3–5 and 14), suggesting that 5mC methylation in the PQSs suppressed the formation of G4s in these regions. Furthermore, the inhibitory effect of 5mC methylation on G4 folding was also examined by testing the ability of these PQSs to bind to the LARK protein, which has been demonstrated to bind G4s, not G4 sequences, with high affinity and specificity [24]. Compared with that of the control, which was not methylated, the binding intensity of LARK with the 5mC-modified PQSs was significantly reduced (Fig. 5i, lanes 6–9). Similar results were obtained with the well-known G4 antibody BG4, which also binds G4s (Fig. 5i, lines 10–13). These results demonstrated that 5mC methylation caused fewer G4s to form in the PQSs.

Besides, the expression of *hPDGFR-β* and *hVEGF* genes was significantly upregulated with treatments of G4 stabilizer pyridostatin (PDS) or methylation inhibitor 5-aza-dC in 293 T and SK-Hep-1 cells (Additional file 1: Fig. S3). This result indicated that either the stabilization of the G4 by PDS or the induction of G4 formation through 5-aza-dC-mediated methylation inhibition consistently activated the expression of *hPDGFR-β* and *hVEGF*.

## Discussion

The formation of G4s is a dynamic process influenced by various biophysical and cellular factors, including ionic conditions [22], G4-binding proteins [24–27], and chromatin structure [28]. 5mC DNA methylation is a critical epigenetic factor of chromatin state and gene transcription, and G4s obtained by various experimental techniques were highly associated with hypomethylation [20, 33, 34, 44]. And the function of G4 on DNA methylation has been reported in several studies [33, 34]. However, the effect of 5mC DNA methylation on G4 formation across the genome-wide remains insufficiently explored.

In this study, a strong negative correlation between G4s and DNA methylation was observed through integrating WGBS and G4 CUT&Tag data of two human cell lines (Fig. 1). In addition to the bioinformatic analyses, this antagonistic relationship was further confirmed by IF experiments in hypomethylated cancer tissues, DNMT1 knockout cells, and cells treated with methylation inhibitors (Figs. 1 and 2). Moreover, when DNMT1 expression was restored by complementarily expressing DNMT1 in DNMT1-KO cells, the signal of G4s correspondingly decreased (Fig. 2). It is worth noting that we found that DNMT1-KO-2 cells have a lower methylation level than DNMT1-KO-1, which may be due to the difference in the cell’s rescue extent or mechanism [54]. The observation that DNMT1-KO-2 had lower methylation and more G4s further proved that lower methylation level can promote G4 formation. All these results verified a conserved negative relationship between DNA methylation and G4 formation in the genome.

Previous study has suggested that G4s are positive elements for CGI hypomethylation [33, 34]. Mechanically, G4s can prevent CpG from being methylated by inhibiting the enzymatic activity of DNMT1 through binding with DNMT1 [33]. From the opposite perspective, the function of DNA methylation on G4 formation in genomic context was unclear. Here, we provide both genome-wide and site-specific evidences demonstrating that DNA methylation inhibits G4 formation. However, DNA methylation may affect the chromatin accessibility [55]. A critical question comes, whether the inhibition of G4 formation by DNA methylation occurs through direct effects or indirectly through effects on chromatin accessibility. Combined ATAC-seq and G4 CUT&Tag analyses showed the suppression of DNA methylation on G4 formation in both open and closed chromatin regions (Fig. 3), indicating that the inhibitory effect is not necessarily coupled with the unwinding of chromatin. Furthermore, in vitro experiments using synthesized DNA oligonucleotides with or without 5mC modification showed that DNA 5mC methylation had a direct inhibitory effect on G4 formation (Fig. 5).

Functionally, G4s have been shown to regulate gene transcription and are primarily enriched in the promoter regions of actively transcribed genes [13, 19, 56]. Conversely, DNA methylation is generally associated with transcriptional repression [50, 57, 58]. By integrating RNA-seq data, we demonstrated that genome-wide hypomethylation induced by DNMT1 knockout enhanced G4 formation and upregulated the transcription of many genes (Fig. 5). And the treatment with a G4 stabilized (PDS) or a DNA methylation inhibitor (5-aza-dC) showed that either stabilization of the G4 in the promoter by G4 stabilizer or the induction of G4 formation through 5-aza-dC-mediated methylation inhibition could activate the expression of *hPDGFR-β* and *hVEGF* containing methylation modification sites in their promoter PQSs. Moreover, consistent with previous findings that methylation inhibited the interaction of SP1 with several G4s and the interaction between VEGF165 and VEGF G4 [37], methylation also inhibited the interaction between the G4 binding protein BmLARK and the *POUM2* G4 (Fig. 4m). All these results suggest that methylation could influence gene transcription by inhibiting G4-protein complex formation, which is a new way for DNA methylation in gene transcription regulation.

**Fig. 6:**
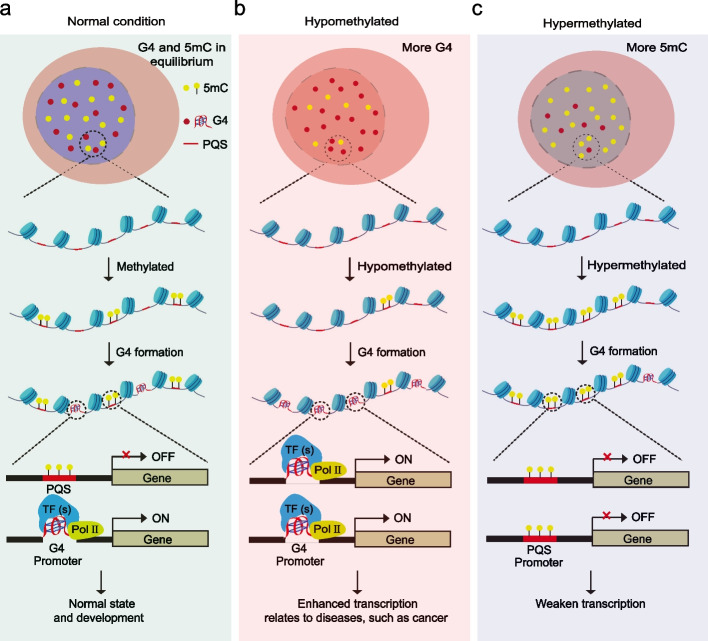
Diagram of the proposed regulatory mechanism of G4s and 5mC methylation in gene transcription. Under normal conditions, global gene transcription is in equilibrium. Genes with PQSs that are not methylated are actively transcribed due to the formation of G4s, while the transcription of genes with methylated PQSs is inhibited (**a**). In some cases, such as cancer, the overall genome is hypomethylated, and more G4s are formed, which results in elevated transcription, especially that of oncogenes (**b**). However, when the cell genome is hypermethylated, G4 formation is inhibited, and overall gene transcription is supressed (**c**)

Based on our findings, we propose a model for gene transcription regulated by DNA methylation and G4s (Fig. 6). Under normal physiological conditions, when PQSs in promoter regions are 5mC methylated, the formation of G4s and the interaction between G4 and G4-binding proteins are suppressed, and gene transcription is maintained at a dynamic balance (Fig. 6a). In certain circumstances, such as during stress or in cancer tissues, global genomic DNA methylation is decreased (Fig. 6b) or increased (Fig. 6c), resulting in more or less G4 and G4-protein complex formation, respectively, which in turn results in fluctuations in gene transcription. This model suggests a regulatory mechanism of gene transcription through the interaction between G4s and DNA methylation in genomes.

There are several novelties and conceptual advancements in this study. Firstly, we provide substantial experimental evidence to demonstrate the inhibitory effect of 5mC methylation on G4 formation at the whole genome level. We know that both 5mC methylation and G4s are epigenetic mechanisms for gene transcription regulation. It has been demonstrated before that G4 has an inhibitory effect on 5mC methylation (33). This study would provide clear and insights into their relationship at the genomic level in the aspect of methylation. Secondly, we also proved that this inhibitory effect of 5mC methylation could occur in both open and closed chromatin states. Thirdly, we found that the inhibitory effect of genomic DNA methylation on gene expression might through affecting genomic G4 formation, revealing a new mechanism of DNA methylation regulating gene transcription.

## Conclusions

G4s have been found to play multiple roles in many cellular processes. Dynamic G4 formation in the genome of specific cells regulates gene transcription and modulates cell growth and development. In this study, an antagonistic correlation between genomic DNA 5mC methylation and G4s was established. Combined WGBS, ATAC-seq, and G4 CUT&Tag omics analyses and in vitro experiments revealed that DNA methylation inhibited G4 formation in both open and closed chromatin states. RNA-seq and G4 CUT&Tag analyses of WT and DNMT1-KO cells also demonstrated that the reduction in the genome-wide methylation level resulted in the formation of more G4s, which then promoted global gene transcription activity. Collectively, these findings help us to better understand the relationship between these two epigenetic regulatory mechanisms and their functions in gene transcription and may have important implications in organism development and human disease.

## Methods

### Cell culture, chemical treatment, and clinical samples

Human 293 T cells (CRL-3216), a derivative of human embryonic kidney 293 cells and SK-Hep-1 human hepatic adenocarcinoma cells (HTB-52), were purchased from American Type Culture Collection (ATCC, VA, USA) and cultured according to the instructions provided with each cell type. All cell lines were maintained in DMEM (11,995,065, Thermo Scientific, MA, USA) supplemented with fetal bovine serum (FBS, 10,099,141, Thermo Scientific, MA, USA), penicillin and streptomycin (15,140,122, Thermo Scientific, MA, USA) at final concentrations of 10%, 100 U/mL, and 100 μg/mL, respectively. The cells were incubated in a 37 °C incubator with 5% CO_2_. Cell line identity was confirmed by STR typing. Testing for mycoplasma was performed periodically to ensure that all cells were free of mycoplasma contamination. The compounds 5-aza-dC (5-Aza-2´-deoxycytidine, A3656, Sigma, Shanghai, China) and RG108 (N-phthalyl-L-tryptophan, HY-13642, MedChemExpress, NJ, USA) were dissolved in DMSO (dimethyl sulfoxide). 293 T and SK-Hep-1 cells grown on glass coverslips were treated with 5 μM 5-aza-dC or 150 μM RG108 in duplicate, and the medium was changed every 24 h for 3 days. PDS was dissolved in ddH_2_O and 10 μM was used to treat the cells for 48 h. Clinical cancerous and noncancerous rectal tissues were collected from a patient diagnosed with rectal cancer at Guangzhou Panyu Central Hospital; the 76-year-old female patient received no preoperative cancer treatment. Immunohistochemical results revealed CK20 (+), CDX-2 (+), BrafV600E (−), MLH1 (+), MSH2 (+), MSH6 (+), PMS2 (+), P53 (approximately 90% +, suggesting a missense mutation), PMS2 (+), Ki-67 (approximately 80% of hot spots), and SATB2 (+). Ethics approval was obtained from the Institutional Research Ethics Committee of Panyu Central Hospital.

### Immunofluorescence staining

Immunofluorescence assays of G4s were conducted as described in previous studies [19]. Cells were seeded on glass coverslips and cultured overnight at 37 °C. Cell fixation was performed by incubating cells with 4% paraformaldehyde for 10 min at room temperature (RT). Then, the cells were permeabilized with 0.2% Triton X-100 in PBS for 10 min at RT. After two rinses with PBS, the cells were blocked for 1 h at 37 °C in a blocking solution composed of 5% bovine serum albumin (BSA) and 5% goat serum in phosphate-buffered saline (PBS). After blocking, the cells were incubated with a BG4-Flag antibody (1:100 diluted in 0.5% goat serum + PBST, MABE917, Merck, Darmstadt, GER) for 1 h at 37 °C, followed by three washes with PBST (5 min each). The cells were incubated with a 1:800 dilution of rabbit anti-Flag antibody (2368, Cell Signaling Technology, MA, USA) for 1 h at 37 °C, followed by three washes with PBST (5 min each). The cells were then incubated with a 1:500 dilution of Alexa Fluor 594-conjugated secondary antibody (A11037, Thermo Fisher, MA, USA) for 45 min at RT, followed by three washes with PBST (5 min each) and a second wash containing DAPI (2.5 μg/mL). Coverslips were mounted on glass slides with ProLong Diamond anti-fade mounting medium (P36970, Thermo Fisher, MA, USA). The immunofluorescence assay of 5mC was conducted as described in a previous study [59]. Similarly, cells grown on coverslips were fixed in 4% paraformaldehyde for 10 min and permeabilized with 0.2% Triton X-100 for 10 min at RT. Then, the cells were treated with 2 M HCl for 30 min at RT and subsequently neutralized for 10 min with 100 mM Tris–HCl buffer, pH 8.5. The cells were blocked in blocking solution (5% BSA and 5% goat serum in PBS) for 1 h at 37 °C. After blocking, the cells were incubated with an anti-5mC antibody (1:200 diluted in 0.5% goat serum + PBST, 39,649, Active Motif, CA, USA) at 4 °C overnight and then washed three times with PBST (5 min each). The cells were then incubated with a 1:500 dilution of Alexa Fluor 488 secondary antibody (A11029, Thermo Fisher, MA, USA) for 45 min at RT and then washed three times with PBST for 5 min each, followed by a wash containing DAPI (2.5 μg/mL). Finally, coverslips were placed on the glass slides with ProLong Diamond anti-fade mounting medium (P36970, Thermo Fisher, MA, USA). Immunofluorescence image acquisition was conducted using an Olympus Fluoview FV1000 confocal microscope. Image Z stacks were obtained using the “pinhole” set to 0.6 Airy units. The same parameters were applied to all the compared samples.

Quantitative statistical analysis of confocal images was performed using ImageJ software. The signals of 100–200 nuclei from three replicates were counted for each condition. The data are shown as the mean ± SEM. The statistical significance of differences between the samples was determined by Student’s *t *test using GraphPad Prism version 5. Fluorescence intensity values were normalized to the nuclear area.

### DNMT1 knockout in cells

To establish knockout cell lines, the genome editing one vector system (PX459) (104,142, Addgene, MA, USA) was used. sgRNAs targeting *DNMT1* were designed using the CRISPR tool (http://crispr.mit.edu). To construct the PX459-gRNA plasmid, short single-strand DNA containing a partial reverse complement of the gRNA sequence was synthesized. After annealing, DNMT1 gRNA with a sticky end containing the *Bbs* I site was formed. The gRNA sequence was annealed to the PX459 plasmid, which was subsequently digested with *Bbs* I to generate the recombinant plasmid PX459-DNMT1-gRNA. The PX459-DNMT1-gRNA plasmid was then transfected into 293 T cells using FuGENE HD Transfection Reagent (E2311, Promega, WI, USA) according to the manufacturer’s instructions. An equal amount of PX459 plasmid DNA was used as a negative control. At 48–72 h after cell transfection, puromycin-containing medium was used to screen for knockout-positive cells. The knockout-positive cell populations were seeded into 96-well plates by limiting dilution analysis. When the cells gradually grew from a 96-well plate to a 6-well plate, genomic DNA was extracted from the cells, and PCR was performed, followed by TA cloning. At least five clones were selected for sequencing verification until homozygous knockout cell lines were obtained for protein expression and functional analysis.

#### Quantitative real-time PCR (qRT-PCR) and Western blotting (WB)

qRT-PCR and WB was performed as described in our previous studies [60]. Total RNA was extracted from treated cells using an Eastep Super Total RNA Extraction Kit (LS1040, Promega, WI, USA). Reverse transcription was conducted using GoScriptTM Reverse Transcription Mix and Random Primers (A2800, Promega, WI, USA) according to the manual. The mRNA levels of individual genes were normalized to the reference gene glyceraldehyde-3-phosphate dehydrogenase (GAPDH), and quantification of transcript levels was performed using the 2^−∆∆Ct^ method. Three times replicates were performed for each gene. The primer sequences are shown in Table S1. Whole-cell lysates were obtained with RIPA buffer, and the protein concentration was measured using a BCA protein assay kit (23,225, Thermo Scientific, MA, USA). A monoclonal antibody against DNMT1 (1:1000; ab188453, Abcam, Cambridge, UK) was used to detect DNMT1. Polyclonal antibodies against His (1:1000; ab9108, Abcam, Cambridge, UK) and GAPDH (1:3000; ab9485, Abcam, Cambridge, UK) were used to detect DNMT1-His and GAPDH, respectively.

### Cell transfection

DNMT1-KO-2 cells were seeded in 12-well culture plates at the desired density and cultured in DMEM (11,995,065, Thermo Fisher, MA, USA) overnight. Cell transfection was performed with FuGENE HD transfection reagent (E2311, Promega, WI, USA) as described previously [24]. Briefly, when the cell density reached 80%, a mixture of 100 μL containing 2 μg of pEGFP (control) or pEGFP-DNMT1, 6 μL of FuGENE HD transfection reagent and an appropriate volume of Opti-MEM Reduced Serum Medium (31,985,070, Thermo Fisher, MA, USA) was prepared and incubated for 15 min at RT. Then, the mixture was added to DNMT1-KO-2 cells in DMEM and cultured for 48 h at 37 °C before G4 immunostaining.

### Frozen sections

Specimens were embedded in freezing compound (Tissue-Tek O.C.T. Compound; Sakura Finetek-USA, USA) and fixed in a precooled specimen disk. Each specimen was frozen at − 20 °C using a Leica CM1950 cryostat (Leica CM1950; Leica Biosystems Nussloch GmbH, GER) and trimmed until the specimen appeared on the cutting surface. The specimens were cut into 5–7 μm sections.

### Circular dichroism (CD)

Wild-type and 5mC-modified oligonucleotides were synthesized by Qingke Biotechnology (Beijing, China). The oligonucleotide sequences are shown in Table S1. Oligonucleotides (5 μM) in 50 mM Tris–HCl (pH 7.5) and 100 mM KCl were heated at 95 °C for 10 min and cooled slowly to RT. CD experiments were performed as described in our previous study [24].

### Electrophoretic mobility shift assay (EMSA)

EMSAs were performed as described in our previous study [60]. Wild-type and 5mC-modified oligonucleotides were labelled with 6-FAM at the 5’ terminus by Qingke Biotechnology and annealed to form G4 probes as described previously [60]. EMSAs were conducted with 2 μM probes. In the binding reaction, 2 μg of purified recombinant LARK protein or 1 μg of BG4 (MABE917, Merck, Darmstadt, GER) was added. The sequences of the oligonucleotide probes used in this study are shown in Table S1. To normalize the band intensity in EMSA assay, the intensity of individual bands, as well as the graph background in blank region, was converted into gray value with ImageJ software. The final intensity of the individual bands was the difference of band gray value minus the blank gray value.

### Bisulfite sequencing PCR (BSP) analysis

*B. mori* genomic DNA was extracted from 2-day-old 2nd instar larvae. Unmethylated cytosines were converted into uracil by using a MethylDetector kit (55,001, Active Motif, CA, USA), whereas methylated cytosines remained unchanged. PCR was then performed with primers designed based on the BmPOUM2 G4 sequence. The PCR products were subsequently sequenced to confirm the 5mC modification sites.

### Cleavage under targets and tagmentation (CUT&Tag)

CUT&Tag was performed essentially as described in a previous study [13], with minor modifications according to the Hyperactive Universal CUT&Tag Assay Kit protocol (TD903, Vazyme, Nanjing, China), each sample analysis was repeated three times with three independently batches of cells and the correlation between three biological replicates was analyzed (Additional file 1: Fig. S4). Briefly, 293 T WT and DNMT1-KO-2 cells (1 × 10^6^) were counted by using a Cellometer Mini (Nexcelom Bioscience, Boston, MA, USA), washed with wash buffer, and immobilized on conA beads with rotation at room temperature for 10 min. The bead-bound cells were incubated (4 °C, rotated overnight) in 50 μL of primary antibody buffer (with 0.05% digitonin) containing a 1:50 dilution of FLAG-tagged BG4 (MABE917, Merck, Darmstadt, GER) or IgG. The next day, the primary antibody buffer was removed, and the cells were washed three times with 200 μL of dig-wash buffer (containing 0.05% digitonin). After washing, the BG4 antibody-incubated cells were resuspended in 50 μL of dig-wash buffer containing a 1:100 dilution of rabbit anti-FLAG antibody (#14,793, CST, MA, USA) and incubated at room temperature for 1 h with slow rotation. The cells were briefly washed with 200 μL of dig-wash buffer three times to remove unbound antibodies. Then, the bead-bound cells were incubated with a 1:100 dilution of donkey anti-rabbit antibody (TD903, Vazyme, Nanjing, China) in 100 μL of dig-wash buffer at room temperature for 30 min with slow rotation. After washing three times with 200 μL of dig-wash buffer, 100 μL of dig-300 buffer with 0.04 μM pA-Tn5 adapter complex was used to resuspend the cells, which were then incubated at room temperature for 1 h with slow rotation. pA-Tn5-bound cells were washed with 500 μL of dig-300 buffer three times, followed by genome excision in 50 μL of 1 × TTBL buffer dilution in dig-300 buffer at 37 °C (800 rpm) using a Thermomixer Comfort. DNA extraction and purification were performed using DNA extraction beads in 100 μL of Buffer L/B (with 5 μL of proteinase K) incubated at 55 °C for 1 h. To generate libraries, purified genomic DNA was amplified with the i5 primer and i7 primer using CAM. The library PCR products were cleaned with VAHTS DNA Clean Beads (#N411, Vazyme, Nanjing, China) and sequenced on an Illumina NovaSeq platform, and 150 bp paired-read reads were generated. The CUT&Tag reads were aligned to the human genome (UCSC hg38) with Bowtie2. The aligned reads were removed with Picard MarkDuplicates, blacklisted regions were removed from the BAM file with bedtools intersect [61], and the deduplicated BAM files were normalized (bins per million mapped reads, BPM) to generate a bigwig file with the bamCoverage command in deepTools 3.3.0 [62]. Peaks were called with MACS2 (v2.2.6) [63]. DNA sequences from the peak were used for motif analyses. Motifs were identified using MEME-ChIP (http://meme-suite.org/tools/meme-chip) with the following parameters: minimum width 15 and maximum width 30. Only the top two significantly enriched motifs with the highest E values are listed in the text.

### Assay for transposase accessible chromatin with high-throughput sequencing (ATAC-seq)

ATAC-seq was performed as previously described [13], with minor modifications according to the High-Sensitivity Open Chromatin Profile Kit 2.0 protocol (N248, Novoprotein, Nanjing, China). Each sample analysis was repeated three times with three independent batches of cells and the correlation between three biological replicates was analyzed (Additional file 1: Fig. S5). Briefly, 5 × 10^4^ cells were collected, washed with cold PBS, and centrifuged at 500 × *g* for 5 min. Then, the cells were lysed with 50 μL of cold lysis buffer (10 mM Tris–HCl, 10 mM NaCl, 3 mM MgCl_2_, 0.1% NP-40, 0.1% Tween-20, 1% digitonin) and incubated on ice for 5 min. Cold wash buffer (950 μL; 10 mM Tris–HCl, 10 mM NaCl, 3 mM MgCl, 0.1% Tween-20) was added to the cells, followed by centrifugation at 500 × *g* for 5 min. The pellet was resuspended in 40 μL of tagmentation buffer (0.3 × PBS, 0.1% Tween-20, 0.01% digitonin, 1 × TD buffer, 1 × pA-Tn5 transposome mix) and incubated at 37 °C for 30 min. The reaction was stopped by the addition of 10 μL of stop buffer at 55 °C for 5 min. The DNA was further purified with 100 μL of Tagment DNA extract beads and eluted with 37 μL of elution buffer. The purified DNA was amplified with 5 × AmpliMix using the N501 and N701 index primers. The purified DNA libraries were size selected with NovoNGS® DNA Clean Beads (N240, Novoprotein, Nanjing, China) and sequenced on the NovaSeq 6000 platform. ATAC-seq reads were aligned to the human genome hg38. The aligned reads were removed with Picard MarkDuplicates, blacklisted regions were removed from the BAM file with bedtools intersect, and the deduplicated BAM files were normalized (bins per million mapped reads, BPM) to generate a bigwig file with the bamCoverage command in deepTools 3.3.0.

### Whole-genome bisulfite sequencing (WGBS)

DNA was isolated using the Cell Culture DNA Midi Kit from QIAGEN (13,343, Qiagen, DUS, GER), and libraries were constructed by E-GENE Co., Ltd (Shenzhen, China). Each sample analysis was repeated three times with three independently batches of cells and the correlation between three biological replicates was analyzed (Additional file 1: Fig. S6). Prior to library construction, 1 µg of sample genomic DNA mixed with spike-in controls (0.3 ng unmethylated lambda DNA) was fragmented to a mean size of approximately 250 bp using a Bioruptor system. After fragmentation, the purified randomly fragmented DNA was subsequently treated with a mixture of T4 DNA polymerase, Klenow fragment, and T4 polynucleotide kinase to repair, blunt, and phosphorylate the ends. The blunt DNA fragments were subsequently 3’ adenylated using Klenow fragment (3’−5’ exo-) (P7010-LC-L, Qiagen, DUS, GER), followed by ligation to adaptors synthesized with 5’-methylcytosine instead of cytosine using T4 DNA Ligase. Then, the DNA was purified using a QIAquick PCR purification kit (28,104, Qiagen, DUS, GER). Then, a ZYMO EZ DNA Methylation-Gold Kit™ (D5005, ZYMO, CA, USA) was used to convert unmethylated cytosine to uracil according to the manufacturer’s instructions. The WGBS libraries were generated by PCR amplification using JumpStart™ Taq DNA Polymerase (D4184-50UN, Sigma, Shanghai, China) and purified using a QIAquick PCR purification kit (28,104, Qiagen, DUS, GER). The WGBS libraries were sequenced using a NovaSeq 6000 analyzer with 150 bp paired-end reads (PE150) according to the manufacturer’s instructions. Low-quality bases and adapter sequences were trimmed by using TrimGalore. The cleaned reads were mapped back to the hg38 genome using BSMAP software version 2.90 [64]. The parameter settings used were “-n 0 -v 0.08 -g 1 -p 48”. Methylation ratios were extracted from BSMAP output (SAM) using a Python script (methratio.py), which is distributed with the BSMAP package. In brief, the methylation level was calculated based on the percentage of methylated cytosine (mC) in the whole genome: site methylation level = 100 × (number of sequences with methylated cytosines (mC)/total number of valid sequences).

### RNA-seq processing and analysis

RNA purity was checked using a NanoPhotometer® spectrophotometer (IMPLEN, CA, USA), and RNA concentration was measured using a Qubit® RNA Assay Kit in a Qubit® 2.0 Fluorometer (Life Technologies, CA, USA). Library preparation and sequencing were performed by Berry Genomics (Beijing, China). Each sample analysis was repeated three times with three independently batches of cells. We confirmed quality using FastQC and processed the raw data with Trim Galore. We aligned the paired-end reads to the human genome (UCSC-hg38) using HISAT2 [65]. A count table file indicating the number of reads per gene in each sample was generated using FeatureCounts.

### Quantification, normalization, and statistical analyses

The DNA methylation level was determined by averaging the methylation levels at the same site across the three replicates. For ATAC-seq and G4 CUT&Tag analyses, the BAM files from the three replicates were merged and normalized by using deepTools-bamCoverage (parameters: binSize 10, normalized using bins per million mapped reads (BPM)). The heatmaps and line plots of DNA methylation, G4, and ATAC signals were generated by using deepTools computeMatrix to calculate the average signal intensities within 10-bp windows for specific DNA regions. For RNA-seq, the counts per million mapped reads (CPM) of each gene were calculated based on the gene expression unit that was normalized for sequencing depth. Differentially expressed genes (DEGs) were identified using DESeq [66], and the genes with a *p*-value < 0.05 and a |log2FC| value > 1 were considered to be DEGs. Statistical analyses were denoted in each figure and figure legend, if applicable.

### Bioinformatics analysis

The whole genome sequence (hg38, https://ftp.ensembl.org/pub/release-107/fasta/homo_sapiens/dna/Homo_sapiens.GRCh38.dna_rm.primary_assembly.fa.gz) was computationally scanned using pqsfinder [67] for identifying PQSs with indicated parameters for the number of guanines in each stack (G-groups), the number of base pairs between G-groups (loop size), and the number of times the loop. The pqsfinder parameters (run_min_len = 3, loop_min_len = 1, loop_max_len = 12, max_defects = 0, min_score = 1) were set to find canonical PQSs (G_3+_N_1-12_)_3_ + G_3+_ [68, 69]. The genomic coordinates of human transcription start sites (TSSs) were downloaded from the UCSC Genome Browser (http://genome.ucsc.edu). computeMatrix was used to split DNA into 10-bp windows to calculate the DNA methylation levels 1 kb upstream and downstream of the TSS [70]. The average DNA methylation ratios of all cytosine sites in each window were used to represent the DNA methylation levels. The 10,000 transcripts with the highest methylation levels and 10,000 transcripts with the lowest methylation levels were selected, and transcripts belonging to the same gene were eliminated to reduce redundancy. These genes were used to calculate the G4 signal strength within 1 kb both upstream and downstream of TSSs at 10-bp windows by using computeMatrix. Heatmaps were generated by the plotHeatmap tool with computeMatrix files, and each heatmap was sorted by decreasing intensity of WGBS signals. Whole-genome bisulfite sequencing datasets for K562 (ENCSR765JPC) and HeLa (ENCSR550RTN) cells were downloaded from ENCODE (https://www.encodeproject.org/), and the BG4 CUT&Tag data for K562 and HeLa cells were obtained from GSE178668 [13].

## Supplementary Information

Additinal file 1: Fig. S1. Immunofluorescence staining of cancerous and noncancerous rectal tissues for the proliferation marker Ki-67. Fig. S2. The 5mC-modified sites in *BmPOUM2* G4 (a), *hPDGFR-β* G4 (b) and *hVEGF* G4 (c). Fig. S3. qRT–PCR analyses of *hPDGFR-β* and *hVEGF* expression in 293 T cells (a, b) or SK-Hep-1 cells (c, d) treated with PDS or 5-aza-dC. Fig. S4. The repeatability of G4 CUT&tag experiments in wildtype 293 T or 293 T-DNMT1-KO cells. (a, c) IGV genome browser tracks of G4 CUT-tag replicates. The reads were aligned to human genome hg38 and normalized by bins per million. (b, d) Correlation plot between every two G4 CUT-tag biological replicates. Fig. S5. The repeatability of ATAC-seq experiments in wildtype 293 T or 293 T-DNMT1-KO cells. (a, c) IGV genome browser tracks of ATAC-seq replicates. (b, d) Correlation plot between every two ATAC-seq biological replicates. Fig. S6. The repeatability of WGBS experiments in wildtype 293 T or 293 T-DNMT1-KO cells. (a) Correlation heat map of WGBS replicates. (b) Circus plot showing genome-wide methylation rate, genes density and CG islands density. Fig. S7. The original images of the WB results shown in Fig. 2k. Fig. S8. The original images of the WB results shown in Fig. 2p. Fig. S9. The original images of the EMSA results shown in Fig. 4i, k, m. Table S1. The primers and oligonucleotide probes used in this study.

## Data Availability

The raw RNA-seq, CUT&Tag, ATAC-seq and WGBS data are available in the GEO repository under the accession number GSE221437 [71]. WGBS data for K562 and HeLa cells were obtained from the ENCODE project under the accession numbers ENCSR765JPC [72] and ENCSR550RTN [73], respectively. G4-CUT&Tag data for K562 and HeLa cells were obtained from the GEO under the accession number GSE178668 [74]. Original western blot (Additional file 1: Fig. S7 and S8) and EMSA images (Additional file 1: Fig. S9) have been added to the Additional file 1. All data supporting the findings of this study are available within the paper and its Additional file 1.
