# Supplementary material for: DNA 5mC methylation inhibits the formation of G-quadruplex structures in the genome
Source: Genome Biol. 2025 Jul 11;26:202. doi: 10.1186/s13059-025-03678-4 (PMC12255034; doi:10.1186/s13059-025-03678-4)
Supplement: Supplementary file 1 — Additinal file 1: Fig. S1. Immunofluorescence staining of cancerous and noncancerous rectal tissues for the proliferation marker Ki-67. Fig. S2. The 5mC-modified sites in BmPOUM2 G4 (a), hPDGFR-β G4 (b) and hVEGF G4 (c). Fig. S3. qRT–PCR analyses of hPDGFR-β and hVEGF expression in 293 T cells (a, b) or SK-Hep-1 cells (c, d) treated with PDS or 5-aza-dC. Fig. S4. The repeatability of G4 CUT&tag experiments in wildtype 293 T or 293 T-DNMT1-KO cells. (a, c) IGV genome browser tracks of G4 CUT-tag replicates. The reads were aligned to human genome hg38 and normalized by bins per million. (b, d) Correlation plot between every two G4 CUT-tag biological replicates. Fig. S5. The repeatability of ATAC-seq experiments in wildtype 293 T or 293 T-DNMT1-KO cells. (a, c) IGV genome browser tracks of ATAC-seq replicates. (b, d) Correlation plot between every two ATAC-seq biological replicates. Fig. S6. The repeatability of WGBS experiments in wildtype 293 T or 293 T-DNMT1-KO cells. (a) Correlation heat map of WGBS replicates. (b) Circus plot showing genome-wide methylation rate, genes density and CG islands density. Fig. S7. The original images of the WB results shown in Fig. 2k. Fig. S8. The original images of the WB results shown in Fig. 2p. Fig. S9. The original images of the EMSA results shown in Fig. 4i, k, m. Table S1. The primers and oligonucleotide probes used in this study. [file 13059_2025_3678_MOESM1_ESM.pdf]

Supplementary information for

DNA 5mC methylation inhibits the formation of G-quadruplex structures in the genome

Kangkang Niu, Lijun Xiang, Xiaojuan Zhang, Xiaoyu Li, Tingting Yao, Jin Li, Chu Zhang, Junpeng Liu, Yuling Peng, Guanfeng Xu, Hui Xiang, Hao Wang, Qisheng Song and Qili Feng

Corresponding author: Qili Feng and Kangkang Niu. Email: qlfeng@scnu.edu.cn. (Q.F.); kkniu@m.scnu.edu.cn (K.N.).

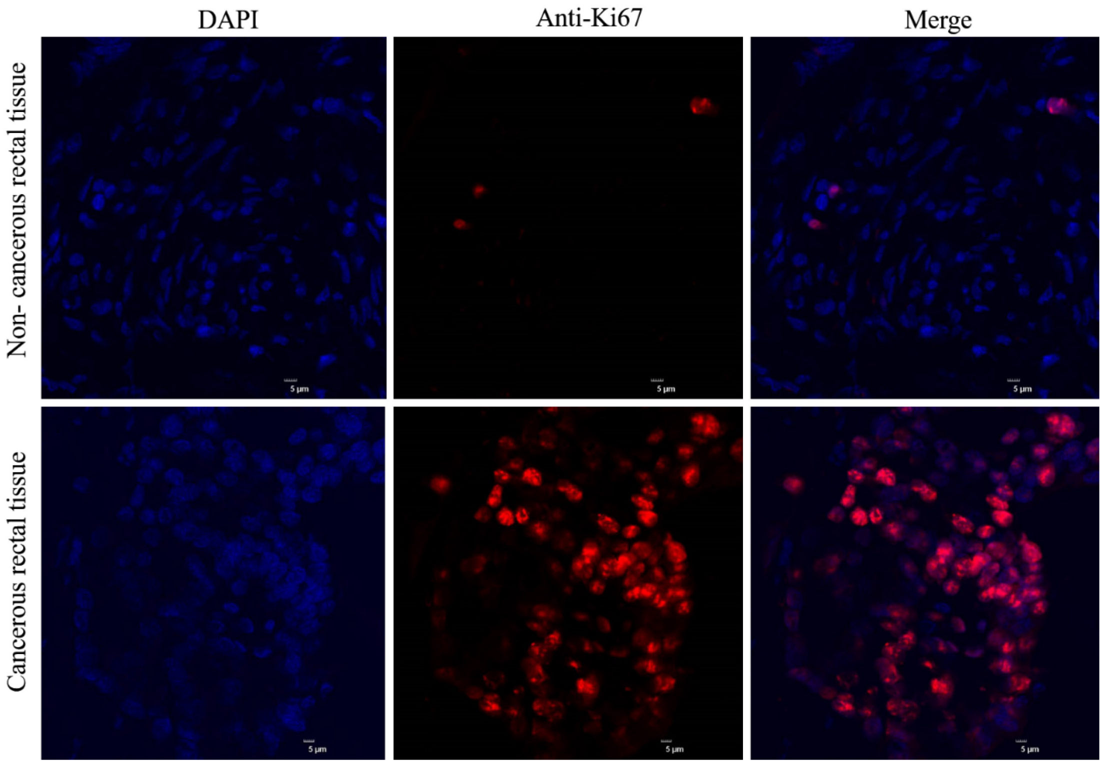

Fig. S1. The immunofluorescence staining of cancerous and non-cancerous rectal tissues for the proliferation marker Ki67.

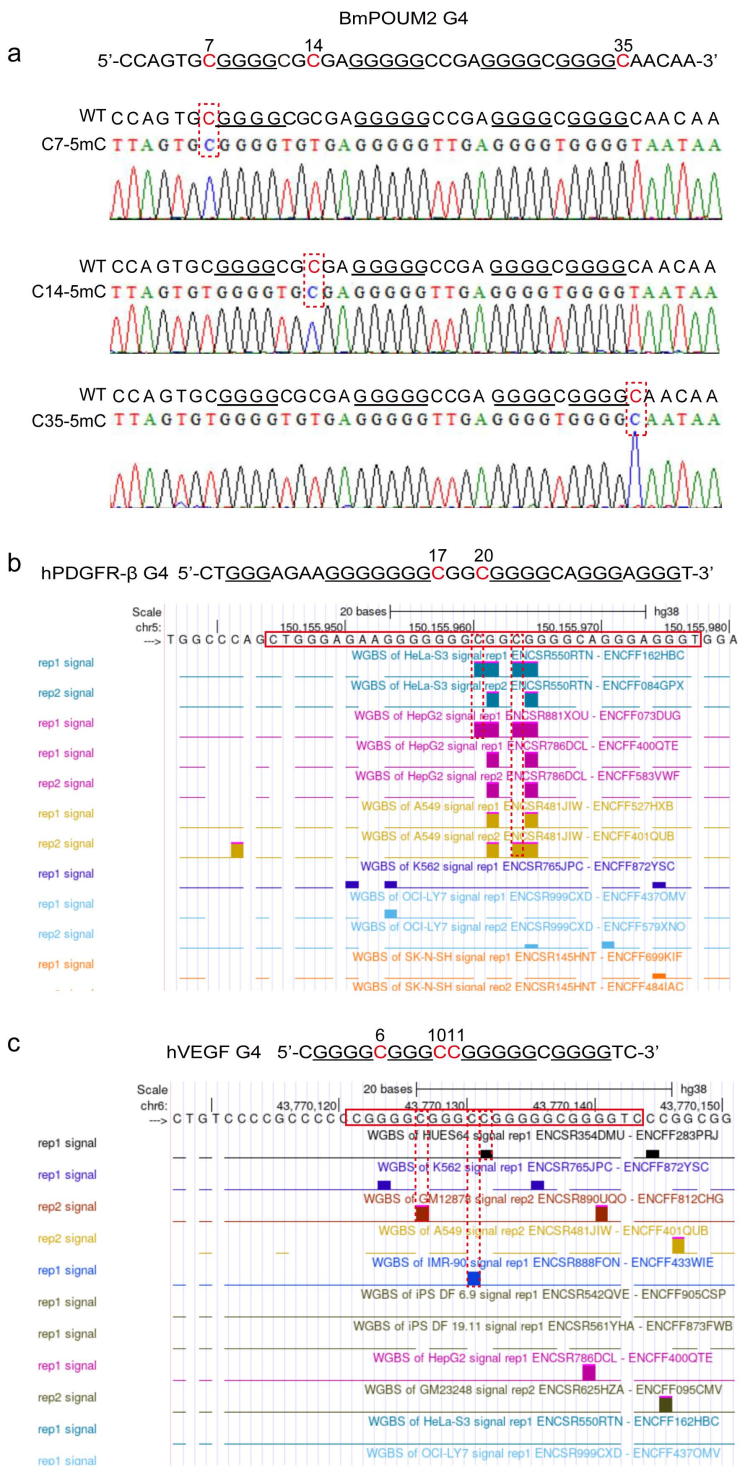

Fig. S2. The 5mC modified sites of BmPOUM2 G4 (a), hPDGFR-β (b) and hVEGF (c). The 5mC modified sites of BmPOUM2 G4 were identified by BSP (Detailed descriptions are provided in the Methods section). The 5mC modified sites information of hPDGFR-β and hVEGF were got from ENCODE database (https://www.encodeproject.org/). The red dotted box indicates the 5mC modified sites.

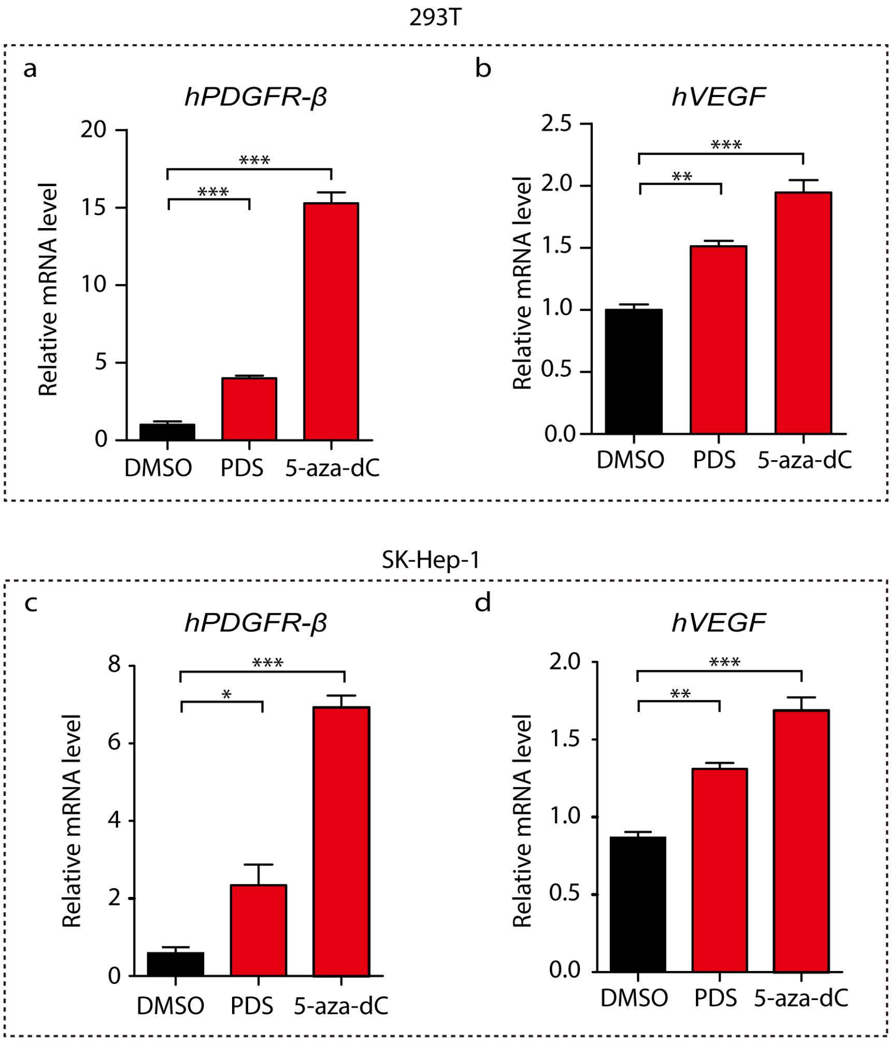

Fig. S3. qRT–PCR analyses of *hPDGFR-β* and *hVEGF* expression in 293T cells (a, b) or SK-Hep-1 cells (c, d) treated with PDS or 5-aza-dC. Data are the mean ± SEM. Statistical significance was determined by Student's t test, *p<0.05, **p<0.01, ***p<0.001.

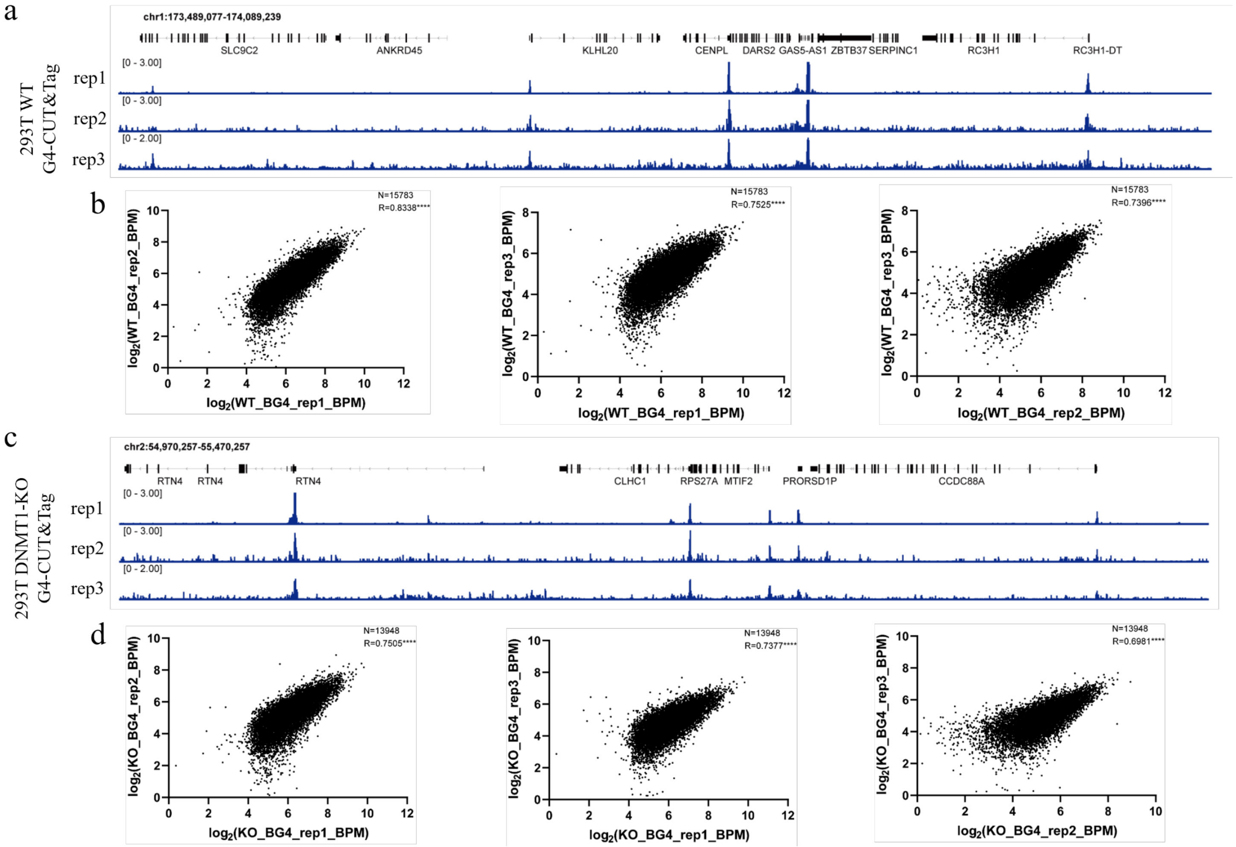

Fig. S4. The repeatability of G4 CUT-tag experiments in wildtype 293T or 293T-DNMT1-KO cells. (a, c) IGV genome browser tracks of G4 CUT-tag replicates. The reads were aligned to human genome hg38 and normalized by bins per million. (b, d) Correlation plot between every two G4 CUT-tag biological replicates.

a

b

c

d

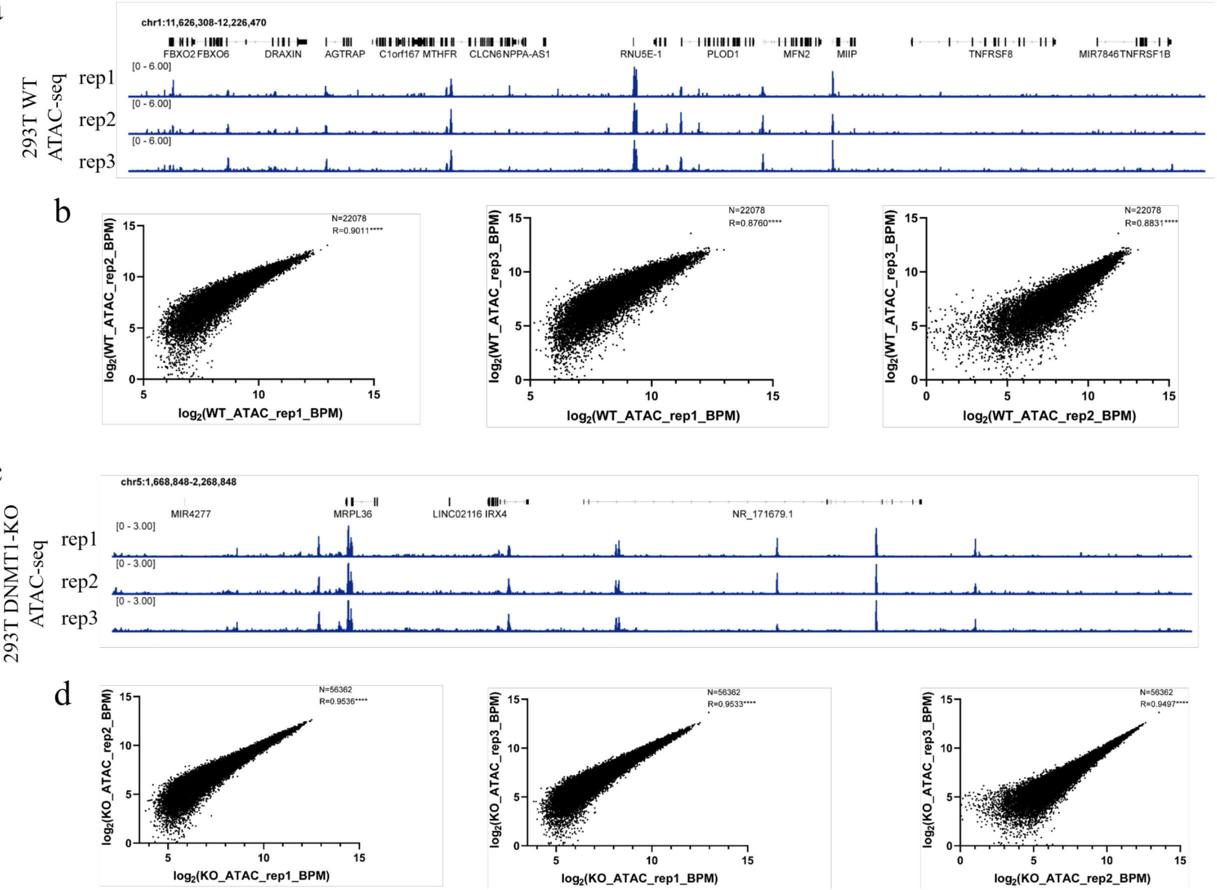

Fig. S5. The repeatability of ATAC-seq experiments in wildtype 293T or 293T-DNMT1-KO cells. (a, c) IGV genome browser tracks of ATAC-seq replicates. (b, d) Correlation plot between every two ATAC-seq biological replicates.

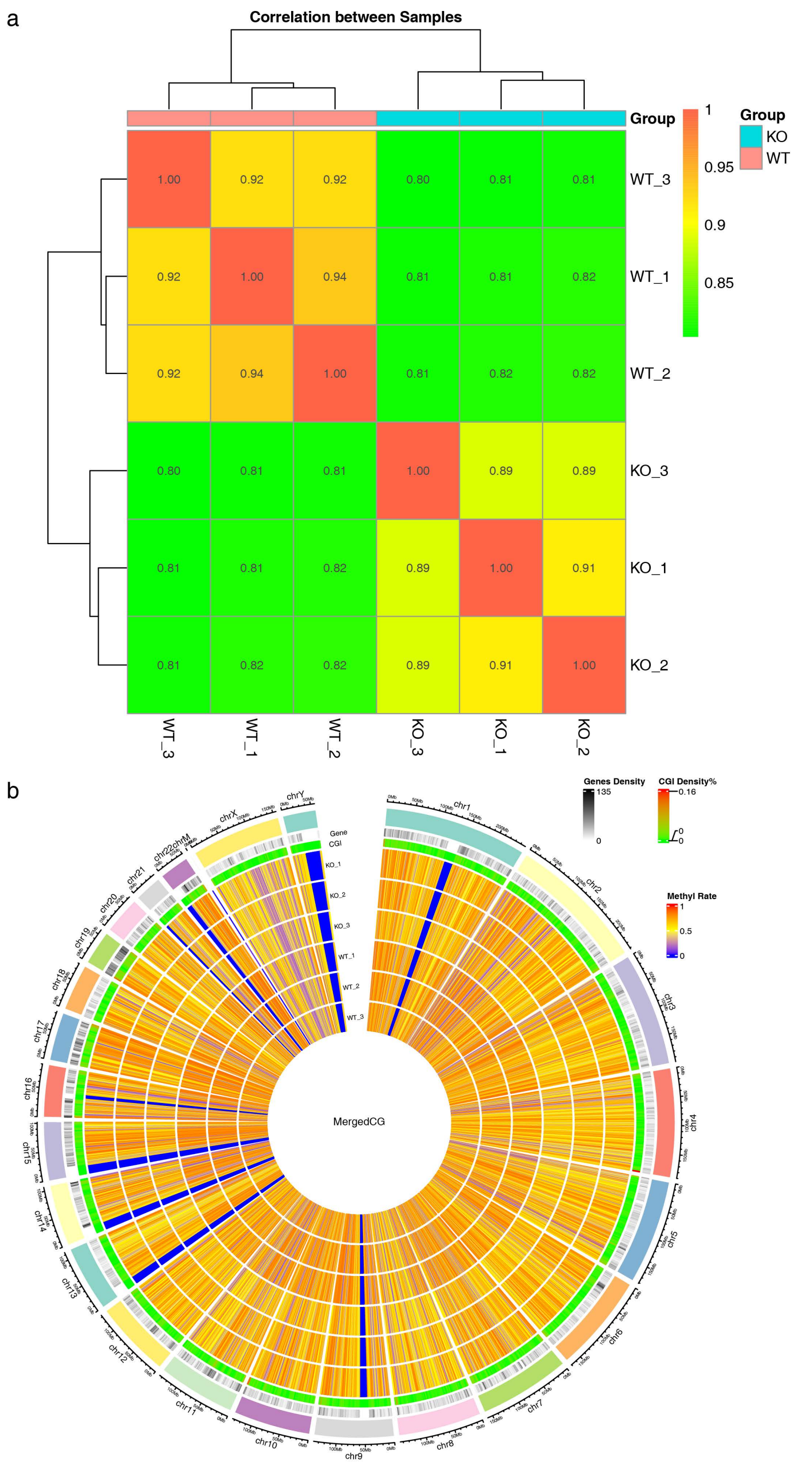

Fig. S6. The repeatability of WGBS experiments in wildtype 293T or 293T-DNMT1-KO cells. (a) Correlation heat map of WGBS replicates. (b) Circus plot showing genome-wide methylation rate, genes density and CG islands density.

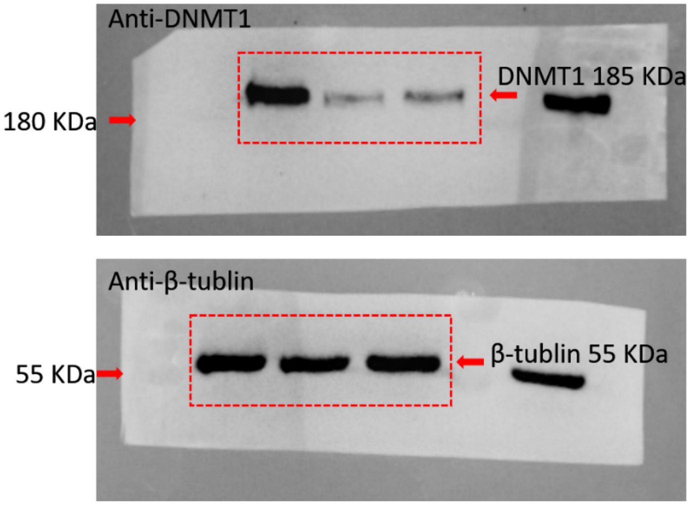

Fig. S7. The original images of WB in Figure 2k.

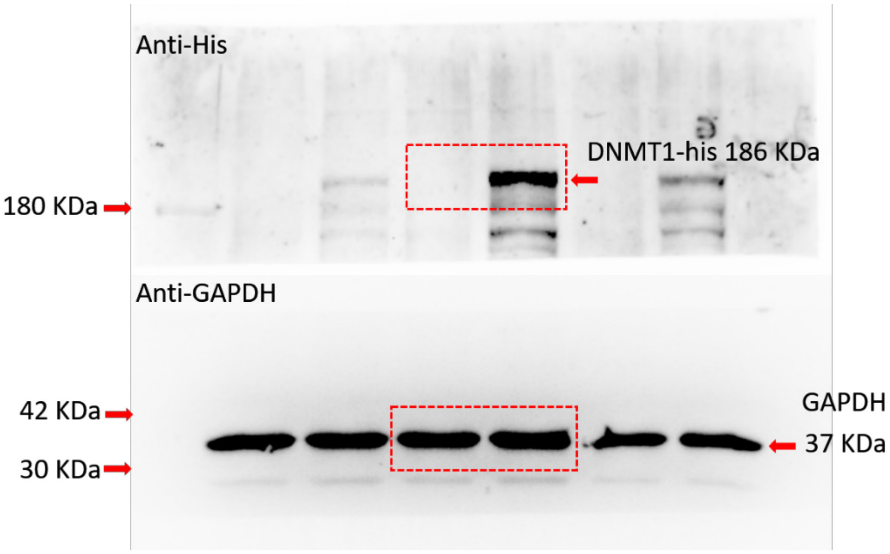

Fig. S8. The original images of WB in Figure 2p.

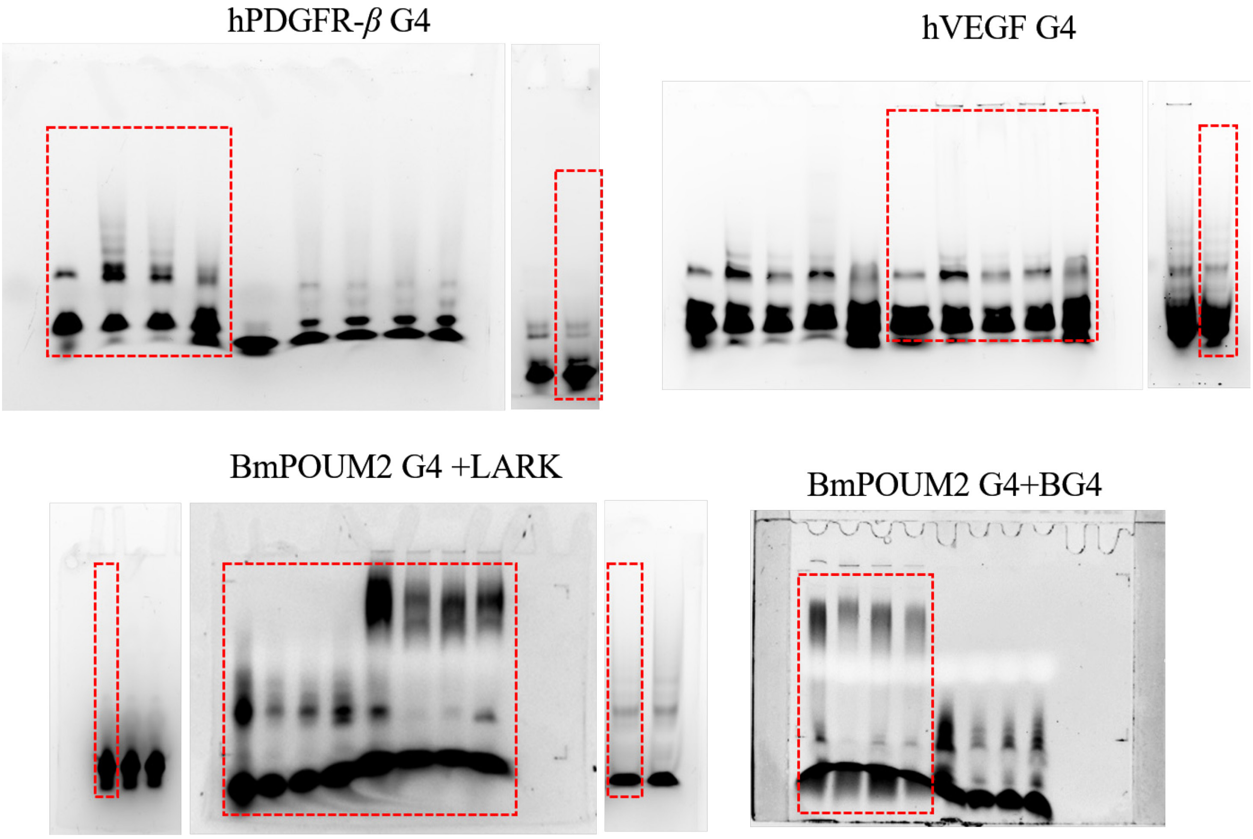

Fig. S9. The original images of EMSA in Figure 4i, k, m.

Table S1. The primers and oligonucleotide probes used in this study.

Sequence name	DNA sequence (the 5mC modified sites were red)
KO-gDNA-F	CACCGCTCTTTCAGACTCTTCCTG
KO-gDNA-R	AAACCAGGAAGAGTCTGAAAGAGC
KO-Test-F	TAGCAGCTGTTGATCGGTGTT
KO-Test-R	AGCCCTAGACAGGGTTTTTATTTAC
DNMT1-qPCR-F	GCAAAATCAGGAACGCGCAC
DNMT1-qPCR-R	CAGTTTCTGTTTGGGTGTTGGTT
BSP-F	GTTTATATGATAAGGAATTGTATTGGGAA
BSP-R	CTCGAACCTTCAACTAACTTATTCTTTC
qPCR-hVEGFA-F	GGTCCCAGGCTGCACCCAT
qPCR-hVEGFA-R	GATGGCTTGAAGATGTACTCGAT
qPCR-hPDGFR-β-F	GCCCAATGAGGGTGACAACG
qPCR-hPDGFR-β-R	GGGCTGTCACAGGAGATGGT
qPCR-GAPDH-F	GATTCCACCCATGGCAAATTC
qPCR-GAPDH-R	CTGGAAGATGGTGATGGGATT
VEGF-WT	CGGGGCGGGCCGGGGGCGGGGTCC
VEGF-C6	CGGGGCGGGCCGGGGGCGGGGTCC
VEGF-C10	CGGGGCGGGCCGGGGGCGGGGTCC
VEGF-C11	CGGGGCGGGCCGGGGGCGGGGTCC
VEGF-C6+10+11	CGGGGCGGGCCGGGGGCGGGGTCC
PDGFR-WT	CTGGGAGAAGGGGGGGCGGCGGGGCAGGGAGGGT
PDGFR-C17	CTGGGAGAAGGGGGGGCGGCGGGGCAGGGAGGGT
PDGFR-C20	CTGGGAGAAGGGGGGGCGGCGGGGCAGGGAGGGT
PDGFR-C17+20	CTGGGAGAAGGGGGGGCGGCGGGGCAGGGAGGGT
POUM2-WT	CCAGTGCGGGGCGCGAGGGGGCCGAGGGGCGGGGCAACAA
POUM2-C7	CCAGTGCGGGGCGCGAGGGGGCCGAGGGGCGGGGCAACAA
POUM2-C14	CCAGTGCGGGGCGCGAGGGGGCCGAGGGGCGGGGCAACAA
POUM2-C35	CCAGTGCGGGGCGCGAGGGGGCCGAGGGGCGGGGCAACAA
POUM2-C7+14+35	CCAGTGCGGGGCGCGAGGGGGCCGAGGGGCGGGGCAACAA
